# Supplementary material for: Interictal neural fragility predicts seizure onset zone and surgical outcomes in drug-resistant epilepsy
Source: PeerJ. 2025 Jul 1;13:e19548. doi: 10.7717/peerj.19548 (PMC12226985; doi:10.7717/peerj.19548)

Supplementary Table 1

| Patient ID | Sex | Age | Diagnostic | MRI positive | CC class | Habitual onset of SOZ | EEGseizures | RF/simulated seizures | RFTC targets | Surgical area | Paological results | Engel Score |
| --- | --- | --- | --- | --- | --- | --- | --- | --- | --- | --- | --- | --- |
| 1 | Male | 28 | Hypoalamic hamarma | 1 | 1 | HB'1-3, HH'1 1-4, HH'2 1-3 |  |  | HB'1-3, HH'1 1-4, HH'2 1-3 | / | / | 1 |
| 2 | Male | 9y2m | Right fron-temporal epilepsy | 0 | 3 | L1'9-13, L2'4-5 |  |  | L1'9-14, L2'1-6 | LITT |  | 1 |
| 3 | Female | 23 | Bilateral paraventricular multiple gray matter hetero-pies | 1 | 4 | / | R2 3-8, R3 4-7, R1 1-2, R4 1-2, R3 1-2, R5 1-2 | R6 1-2, IL '3-5 | R1 1-3, R2 1-3, 4-8, R3 1-3, 4-8, 11T2, R4 1-3, 5-7, R5 1-3, 9T2, R6 1-3, 7-9, IL '3-5 | / | / | 1 |
| 4 | Male | 49 | Bilateral paraventricular multiple gray matter hetero-pies | 1 | 4 | HT: 1-3 | HT: 11-12 → HT: 9-12, L1: 8-10 | HT: 1-2 | HT 1-5, 11-12; L1 4-5; L2 1-3; L3 1-3, 6-8; HB'1-2, 4-6; R1'1-2, 7-8; | / | / | 1 |
| 5 | Female | 48 | Left hippocampal sclerosis | 0 | 2 | HP: 1-4 | HP: 1-4 |  | HP 1-3, A 1-3, HBa 1-2, HBp 1-2, IL 1-4 | / |  | 1 |
| 6 | Male | 20 | Left fron-temporal epilepsy | 0 | 3 | A1-3, AL1-4 , HP1-4, HB1-3, HTa1-2 | / | / | A 1-3, AL 1-4, HP 1-4, HB 1-3, HTa 1-2 | / | / | 1 |
| 7 | Male | 29 | Left frontal epilepsy | 0 | 3 | SMA2: 5-7, 10-12 | SMA2: 5-7, 10-12 |  | SMA2: 5-7, 10-12 | LITT | / | 4 |
| 8 | Female | 33 | Right parietal epilepsy | 0 | 3 | / | / | / | P1 '1-3, PCC' 1-2, P3 '1-3, P3' 5-6, P1 '5-7, 10-12, P2' 6-11 | / | / | 1 |
| 9 | Male | 28 | Left temporal epilepsy | 0 | 2 | P1:1-3; P3:1-2 | ; P2:4-6 |  | P1:1-3; P2:4-6; P3:1-2 | / | / | 1 |
| 10 | Female | 16 | Right temporal & occipital epilepsy | 0 | 3 | 02 '5-13, 03' 1-12, HP, 13-17 HP '2-4, 6-9 |  |  | 01'1-3 O2'5-13 O3'1-12, HP'1-10 13-16 | Right occipital and right partial hippocampus | Cortical dysplasia | 1 |
| 11 | Female | 36 | Bilateral Fron-temporal epilepsy | 0 | 3 | HB '1-4, HP, 1-6, IL' 2-4 |  |  | HB '1-4, HP, 1-6, IL' 2-4 | / | / | 1 |
| 12 | Female | 41 | Right temporal epilepsy | 0 | 2 | HT '6-10, HB' 3-8 or HP '1-3, HT' 1-2, 6T0, A '1-4, HB' 1-2, IL '1-4 |  |  | HT '6-10, HB' 3-8 or HP '1-3, HT' 1-2, 6T0, A '1-4, HB' 1-2, IL '1-4 | / | / | 1 |
| 13 | Female | 15 | Left fron-temporal epilepsy | 0 | 3 | HP 1-3 (1-4), HB 1-3, HT 1-3, A 1-3 |  |  | HP 1-3 (1-4), HB 1-3, HT 1-3, A 1-3 ACC 4-9, OF 7-9, IFL 1-5, | / | / | 4 |
| 14 | Female | 5y10m | Left frontal epilepsy | 0 | 3 | A4:1-3, A3:1-2 HP1-3 |  |  | A2 1-3, A3 1-5 , A4 1-5, HP 1-3 | / | / | 3 |
| 15 | Female | 12 | Right temporal epilepsy | 0 | 2 | HP 1-4, HB 1-3 , A 1-3, IL 1-3 |  |  | A 1-3, 6-8, HP 1-4, HB 1-3, HT 1-5, ACC 1-2, SMA 1-2, OF 1-3, 9-10, IL 1-3, aMCC 1-2 | LITT | / | 1 |
| 16 | Male | 23 | Left frontal epilepsy | 0 | 3 | AMCC1-3, IL4-6, 10-12, OF12-17 pMCC6-11, L1:8-10, aMCC6-12 | / | / | AMCC1-3, IL4-6, 10-12, OF12-17 pMCC6-11, L1:8-10, aMCC6-12 | LITT | / | 1 |
| 17 | Female | 29 | Right fron-temporal epilepsy | 0 |  | HP 1-3, pMCC 12-15, AL 3-5 |  |  | HP 1-3, pMCC 12-15, AL 3-5, aMCC 8-12, HB 6-9, AS 5-8 | LITT | / | 2 |
| 18 | Male |  | Left insular epilepsy & Right temporal epilepsy | 0 | 3 | IL1-3 HP1-3, HB1-3, A1-3, HT1-2 ; HP '1-2 |  |  | IL1-3 HP '1-2 | / | / | 4 |
| 19 | Male | 14 | Left frontal epilepsy | 1 | 1 | ACC 2-4, 6-8, OFG 2-4, F1 1-3, 5-7, 10-12, F2 1-3, 6-10 IL 1-3 |  |  | ACC 2-4, 6-8, OFG 2-4, F1 1-3, 5-7, 10-12, F2 1-3, 6-10 IL 1-3 | RNS | / | 4 |
| 20 | Female | 18 | Left parietal epilepsy | 0 | 3 | MCC Z1 1-3, PCC 1-3, 8-12 |  |  | MCC Z1 1-3, PCC 1-3, 8-12 | RNS | / | 4 |
| 21 | Male | 21 | Left fron-temporal epilepsy | 0 | 3 | HB1-3, HP1-3, IL1-3 |  |  | HB1-3, HP1-3, IL1-3 | Left anterior temporal lobe, hippocampus, amygdala | Cortical dysplasia | 1 |
| 22 | Male | 29 | Right fron-temporal epilepsy | 0 | 2 | HP'1-4 |  |  | HP 'electrode 1.2.3.4.7.8.9.10; | Right anterior temporal lobe+right hippocampus, amygdala resection | FCDIb | 1 |
| 23 | Female | 30 | Right frontal epilepsy (FCD) | 1 | 1 | IL'1A8-10, ACC'A 6-8 |  |  | IL'1（8.9.10.14.15）; IL'2 （8.9.10）; ACC '（6.7.8）; HP '（1.2.3.4）; HB '（1.2.3.4）; MCC '（3.4.7.8）; F'（13.14.15）; ACC（6.7.8.9 ） | Excision of lesion of right frontal brain | FCDIb | 1 |
| 24 | Male | 31y | Left temporal epilepsy | 0 | 2 | A1-4, HP1-3 | / | / | / | Left anterior temporal lobe, hippocampus, amygdala | Hippocampal sclerosis | 1 |
| 25 | Male | 13y7m | Left frontal epilepsy | 0 | 3 | F01 6-10, OFC 1-6, SSMA 4-8 |  |  | MCC 11-15 SSMA 4-8 FO1 6-10 OFC '13-15 | Left frontal lobe anterior portion (left middle frontal gyrus epilepsy+left superior frontal gyrus epilepsy+left superior frontal gyrus epilepsy+left superior frontal gyrus epilepsy) | Cortical dysplasia | 1 |
| 26 | Male | 31 | Left frontal epilepsy | 0 | 3 | F5:1-3 F1:1-3, MCC 12-14, F2:7-8, 1-2 |  |  | F5:1-3 F1:1-3, MCC 12-14, F2:7-8, 1-2 | Left frontal auxiliary mo-r area | Cortical dysplasia | 1 |
| 27 | Male | 37 | Right temporal epilepsy | 0 | 2 | HP'1-3 |  |  | / | Right anterior temporal lobe, right hippocampus, amygdala | Hippocampal sclerosis | 1 |
| 28 | Male | 32 | Left temporal epilepsy & Right frontal epilepsy | 0 | 3 | HB 1-3, HP 1-3, OF 1-2, HB 6-9 | HB 1-3, HP1-2, or ACC '1-5 |  | HB 1-3, 6-9, HP 1-3, ACC '1-10, OF 1-2 | Right frontal softening lesion, left anterior temporal lobe, hippocampus, amygdala | Cortical dysplasia | 1 |
| 29 | Female | 36 | Left occipital epilepsy | 0 | 3 | 0 1-3, 5-7 |  |  | / | Left occipital lobe epileptic focus | Cortical dysplasia | 1 |
| 30 | Male | 5y1m | Right frontal epilepsy | 0 | 3 | L2 '6-9, SMA' 8-10 |  |  | L2 '5-10 | Right frontal lobe | Cortical dysplasia | 3 |

**Supplementary Table 2a. Individual Average Fragility of SOZE/NSOZE**

| Patients ID | Outcome | Average Fragility of SOZE | Average Fragility of NSOZE |
| --- | --- | --- | --- |
| 1 | 1 | 0.10 | 0.17 |
| 2 | 1 | 0.57 | 0.44 |
| 3 | 1 | 0.44 | 0.48 |
| 4 | 1 | 0.49 | 0.35 |
| 5 | 1 | 0.68 | 0.30 |
| 6 | 1 | 0.56 | 0.33 |
| 7 | 1 | 0.55 | 0.39 |
| 8 | 1 | 0.63 | 0.52 |
| 9 | 1 | 0.57 | 0.39 |
| 10 | 1 | 0.63 | 0.38 |
| 11 | 1 | 0.64 | 0.34 |
| 12 | 1 | 0.46 | 0.26 |
| 13 | 1 | 0.40 | 0.36 |
| 14 | 0 | 0.16 | 0.11 |
| 15 | 0 | 0.32 | 0.14 |
| 16 | 0 | 0.47 | 0.30 |
| 17 | 0 | 0.45 | 0.36 |
| 18 | 0 | 0.41 | 0.32 |
| 19 | 0 | 0.38 | 0.33 |
| 20 | 0 | 0.73 | 0.27 |
| 21 | 1 | 0.43 | 0.37 |
| 22 | 1 | 0.55 | 0.36 |
| 23 | 1 | 0.55 | 0.50 |
| 24 | 1 | 0.44 | 0.42 |
| 25 | 1 | 0.52 | 0.40 |
| 26 | 1 | 0.59 | 0.49 |
| 27 | 1 | 0.43 | 0.38 |
| 28 | 1 | 0.45 | 0.35 |
| 29 | 1 | 0.71 | 0.47 |
| 30 | 0 | 0.50 | 0.34 |

**Supplementary Tablec 2b. Average Fragility SOZE vs NSOZE in Patients with Different Outcome**

| Outcome | t-statistic | p-value |
| --- | --- | --- |
| 0 | 2.816511157 | 0.025902645 |
| 1 | 6.169983647 | 4.03E-06 |

Supplementary Table2a Individual average fragility of SOZ/NSOZ. Average fragility of SOZ/NSOZ of each patient displayed in the table. Table2b displays paired t test results of SOZ vs NSOZ in patients with different outcome, which demonstrates that fragility differential distribution between SOZ and NSOZ in the success outcome patients more significantly compared to that in failed outcome patients. The t-statistic indicates the ratio of the difference between the means of two groups to the standard error of that difference. The p-value represents the probability that the observed difference occurred by chance, with values below 0.05 indicating statistical significance.

Abbreviations: Outcome = Patients achieved seizure free (success, 1) or not (failed, 0), SOZE seizure onset zone defined as the SEEG channels exhibiting the earliest electrophysiological changes at seizure onset, NSOZE extra seizure onset zone

**Supplementary3**

We constructed a spatiotemporal heatmap to capture the dynamic fragility changes across 13,975 sampling points for each electrode. This heatmap was subsequently integrated with a random forest classifier（Supplementary Figure1. Random Forest Classifier for SOZ Prediction） to predict the seizure onset zone (SOZ). For patients with successful outcomes validated by RFTC or surgery, the target SOZ channels were labeled as 1, while the remaining channels (NSOZ) were labeled as 0. In contrast, for patients with unsuccessful outcomes post-RFTC or surgery, channels corresponding to SOZ supported by EEG evidence were labeled as 1, and all other channels were labeled as 0. In individual SOZ predictions, most electrodes are classified as NSOZ, resulting in a severe class imbalance between SOZ and NSOZ categories. To address this imbalance, we applied the Synthetic Minority Over-sampling Technique (SMOTE), which combines oversampling of the minority class and undersampling of the majority class. The balanced random forest algorithm was implemented using the open-source Python toolbox imbalanced-learn (https://github.com/scikit-learn-contrib/imbalanced-learn) and was adapted for this study. To evaluate the model's performance, we used a five-fold cross-validation method to generate Receiver Operating Characteristic (ROC) curves and calculate the Area Under the Curve (AUC). Additionally, we assessed the model's precision, recall, and overall accuracy. The model achieved an overall accuracy of 0.98, with a mean accuracy of 0.976 across the five-fold cross-validation. This indicates consistent and excellent performance across different data splits, with accuracy consistently exceeding 97%. The AUC for the ROC curve was 0.99, demonstrating high discriminative power. The model's precision and recall for SOZ prediction were 0.96 and 0.94, respectively, and for NSOZ prediction, they were 0.98 and 0.99, indicating satisfactory predictive performance for both categories.


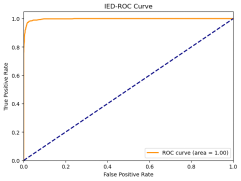

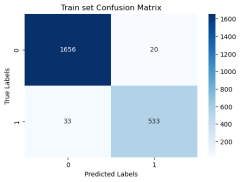


**Supplementary Figure1. Random Forest Classifier for SOZ Prediction**

**Supplementary Table 3.Individual SOZ prediction for SOZE and SOZC**

| Patients ID | SEEG Eletrodes | SOZE Eletrodes | SOZE | | SOZC | | RFTC | Surgery | Engel Score | MRI | CC |
| --- | --- | --- | --- | --- | --- | --- | --- | --- | --- | --- | --- |
|  |  |  | precision | recall | precision | recall |  |  |  |  |  |
| 1 | 104 | 10 | 1.00 | 1.00 | 1.00 | 1.00 | 1 | 0 | 1 | 1 | 1 |
| 2 | 120 | 7 | 1.00 | 1.00 | 1.00 | 1.00 | 1 | 0.5 | 1 | 0 | 3 |
| 3 | 122 | 19 | 0.58 | 1.00 | 1.00 | 1.00 | 1 | 0 | 1 | 1 | 4 |
| 4 | 120 | 2 | 0.36 | 1.00 | 0.82 | 1.00 | 1 | 0 | 1 | 1 | 4 |
| 5 | 155 | 4 | 0.18 | 0.75 | 1.00 | 1.00 | 1 | 0 | 1 | 0 | 2 |
| 6 | 133 | 16 | 0.56 | 0.63 | 1.00 | 1.00 | 1 | 0 | 1 | 0 | 3 |
| 7 | 128 | 6 | 1.00 | 1.00 | 0.57 | 0.67 | 1 | 0.5 | 4 | 0 | 3 |
| 8 | 102 | 11 | 0.50 | 1.00 | 0.95 | 1.00 | 1 | 0 | 1 | 0 | 3 |
| 9 | 115 | 5 | 0.71 | 1.00 | 1.00 | 0.88 | 1 | 0 | 1 | 0 | 2 |
| 10 | 124 | 31 | 0.73 | 0.97 | 0.93 | 1.00 | 1 | 1 | 1 | 0 | 3 |
| 11 | 137 | 8 | 0.37 | 0.95 | 0.90 | 1.00 | 1 | 0 | 1 | 0 | 3 |
| 12 | 95 | 26 | 0.67 | 1.00 | 1.00 | 1.00 | 1 | 0 | 1 | 0 | 2 |
| 13 | 104 | 13 | 1.00 | 1.00 | 1.00 | 0.13 | 1 | 0 | 4 | 0 | 3 |
| 14 | 91 | 7 | 0.91 | 1.00 | 1.00 | 1.00 | 1 | 0 | 3 | 0 | 3 |
| 15 | 98 | 9 | 0.26 | 1.00 | 0.94 | 1.00 | 1 | 0.5 | 1 | 0 | 2 |
| 16 | 103 | 36 | 1.00 | 1.00 | 1.00 | 1.00 | 1 | 0.5 | 1 | 0 | 3 |
| 17 | 120 | 3 | 0.06 | 1.00 | 0.86 | 0.97 | 1 | 0.5 | 2 | 0 | 3 |
| 18 | 154 | 17 | 1.00 | 1.00 | 0.24 | 1.00 | 1 | 0 | 4 | 0 | 3 |
| 19 | 106 | 23 | 1.00 | 1.00 | 1.00 | 0.44 | 1 | 2 | 4 | 1 | 1 |
| 20 | 117 | 11 | 0.93 | 1.00 | 1.00 | 1.00 | 1 | 2 | 4 | 0 | 3 |
| 21 | 133 | 4 | 0.79 | 0.86 | 0.97 | 0.97 | 1 | 1 | 1 | 0 | 3 |
| 22 | 120 | 10 | 0.82 | 0.90 | 1.00 | 0.92 | 1 | 1 | 1 | 0 | 2 |
| 23 | 131 | 6 | 1.00 | 1.00 | 1.00 | 1.00 | 1 | 1 | 1 | 1 | 1 |
| 24 | 111 | 7 | 1.00 | 1.00 | 1.00 | 1.00 | 0 | 1 | 1 | 0 | 2 |
| 25 | 117 | 16 | 0.19 | 1.00 | 0.97 | 1.00 | 1 | 1 | 1 | 0 | 3 |
| 26 | 135 | 13 | 0.50 | 0.44 | 1.00 | 0.88 | 1 | 1 | 1 | 0 | 3 |
| 27 | 118 | 3 | 1.00 | 1.00 | 1.00 | 0.48 | 0 | 1 | 1 | 0 | 2 |
| 28 | 112 | 20 | 1.00 | 1.00 | 1.00 | 1.00 | 1 | 1 | 1 | 0 | 3 |
| 29 | 108 | 6 | 1.00 | 1.00 | 1.00 | 1.00 | 0 | 1 | 1 | 0 | 3 |
| 30 | 100 | 7 | 1.00 | 0.96 | 1.00 | 0.76 | 1 | 1 | 3 | 0 | 3 |

Supplementary Table3 Individual SOZ prediction for SOZE and SOZC . Patient data and prediction (precision/recall) results for SOZE and SOZC.

Abbreviations: SEEG Eletrodes = Number of electrodes included in SEEG implantation, SOZE Eletrodes = Number of electrodes included in seizure onset zone identified with ictal EEG support, SOZC Eletrodes = Number of electrodes included in RFTC/LITT/Surgery targets, RFTC = Patients received RFTC ablation (1) or not (0), Surgery = Patients received LITT(0.5), resection surgery (1) or no surgery (0), MRI=Magnetic Resonance Imaging display leision(1) or not(0), CC(1-4)=Clinical Complexity(I-IV), RFTC Radiofrequency thermocoagulation, SEEG Stereotactic-electroencephalography, LITT Laser interstitial thermal therapy.

**Supplementary Table 4**. Supplementary for Fig3 Model prediction for SOZE in Patients with Different Outcome

| Outcome Group | Metric | Min | 25% | 50% (Median) | 75% | Max |
| --- | --- | --- | --- | --- | --- | --- |
| Failure | recall | 0.96 | 1.00 | 1.00 | 1.00 | 1.00 |
|  | precision | 0.06 | 0.92 | 1.00 | 1.00 | 1.00 |
| Success | recall | 0.44 | 0.95 | 1.00 | 1.00 | 1.00 |
|  | precision | 0.18 | 0.50 | 0.72 | 1.00 | 1.00 |

**Supplementary Table 4.** Supplementary for Fig3 SOZ prediction for SOZE . This table presents the quartile statistics of the model’s concordance metrics (SOZE recall and SOZE precision) for predicting the SOZE across different Engel score groups.Note that precision, and recall results are based on n=22 interictal SEEG data snapshots (13975 sampling points)(from seizure-free group of 22 patients) and n=8 interictal SEEG data snapshots (from non-seizure-free group of 8 patients). Note that box plot minima = minimum value in the data, maxima = maximum value in the data, centre = median, upper boundary of box is 75th percentile, lower boundary of box is 25th percentile, lower boundary of whisker is defined as 25th percentile minus 1.5 times interquartile range (Q3–Q1), i.e. Q1–1.5*(Q3−Q1) and upperboundary of whisker is defifined as 75th percentile plus 1.5 times interquartile range (Q3−Q1), i.e. Q3 + 1.5*(Q3−Q1). Black dot indicates outlier.

Abbreviations: SOZE SOZ seizure onset zone identified with ictal EEG support.

**Supplementary Table 5.** Supplementary for Fig4 Model prediction for SOZC in Patients with Different Outcome

| Engel Score | Metric | Min | 25% | 50% (Median) | 75% | Max |
| --- | --- | --- | --- | --- | --- | --- |
| 1 | recall | 0.48 | 1.00 | 1.00 | 1.00 | 1.00 |
|  | precision | 0.82 | 0.97 | 1.00 | 1.00 | 1.00 |
| 2 | recall | 0.97 | 0.97 | 0.97 | 0.97 | 0.97 |
|  | precision | 0.86 | 0.86 | 0.86 | 0.86 | 0.86 |
| 3 | recall | 0.76 | 0.82 | 0.88 | 0.94 | 1.00 |
|  | precision | 1.00 | 1.00 | 1.00 | 1.00 | 1.00 |
| 4 | recall | 0.13 | 0.44 | 0.67 | 1.00 | 1.00 |
|  | precision | 0.24 | 0.57 | 1.00 | 1.00 | 1.00 |

**Supplementary Table 5.** Supplementary for Fig5 Model prediction for SOZC. This table presents the quartile statistics of the model’s concordance metrics (SOZC recall and SOZC precision) for predicting the SOZ against the clinical therapeutic target SOZC across different Engel score groups.

Note that box plot minima = minimum value in the data, maxima = maximum value in the data, centre = median, upper boundary of box is 75th percentile, lower boundary of box is 25th percentile, lower boundary of whisker is defined as 25th percentile minus 1.5 times interquartile range (Q3–Q1), i.e. Q1–1.5*(Q3−Q1) and upperboundary of whisker is defifined as 75th percentile plus 1.5 times interquartile range (Q3−Q1), i.e. Q3 + 1.5*(Q3−Q1). Black dot indicates outlier.

Abbreviations: SOZC SOZ seizure onset zone identified with clinical RFTC/LITT/Surgery.

**Supplementary Table 6.** Supplementary for Fig5b The Statistical Characteristics of Each Variable in The Regression Model

|  | Coefficient | P-Value | 95% CI Lower Bound | 95% CI Upper Bound |
| --- | --- | --- | --- | --- |
| const | 4.09 | 0.05 | -0.01 | 8.20 |
| SOZE recall | 0.85 | 0.48 | -1.62 | 3.33 |
| SOZE precision | 0.76 | 0.18 | -0.37 | 1.89 |
| SOZC recall | -2.66 | 0.00 | -4.17 | -1.16 |
| SOZC precision | -3.52 | 0.00 | -5.55 | -1.49 |
| RFTC | 1.20 | 0.03 | 0.15 | 2.24 |
| Surgery | 0.66 | 0.03 | 0.05 | 1.26 |
| MRI | -0.12 | 0.77 | -0.96 | 0.72 |
| CC | 0.15 | 0.46 | -0.27 | 0.58 |

**Supplementary Table 5.** Supplementary for Fig5b The Statistical Characteristics of Each Variable in The Regression Model：The table sequentially presents each variable's regression coefficient, p-value, and 95% confidence interval.

Abbreviations: SOZE recall = SOZ prediction model recall for SOZE, SOZE precision = SOZ prediction model precision for SOZE, SOZE = Seizure onset zone identified with ictal EEG support, SOZC recall = SOZ prediction model recall for SOZC, SOZC precision = SOZ prediction model precision for SOZC,SOZC = Seizure onset zone included in RFTC/LITT/Surgery targets, RFTC Radiofrequency thermocoagulation, MRI Magnetic Resonance Imaging, CC Clinical Complexity

**Results Summary (also refer to Supplementary Table 1)**

SEEG seizures data and interictal SEEG fragility heatmap are displayed below.

Patients ID 1


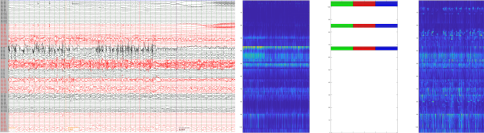


Patients ID 2


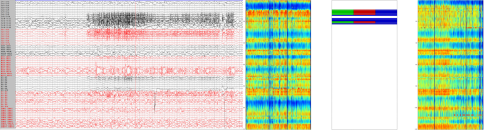


Patients ID 3


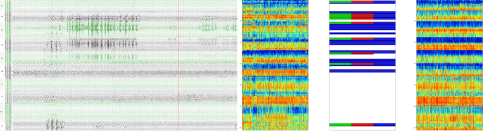


Patients ID 4


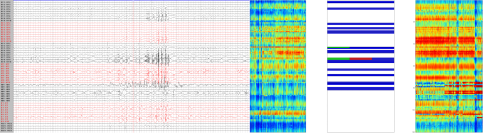


Patients ID 5


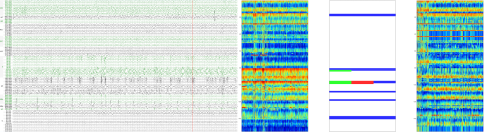


Patients ID 6


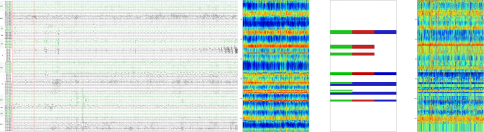


Patients ID 7


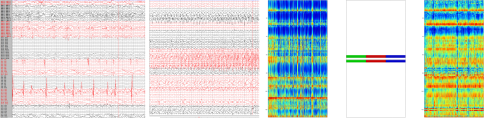


Patients ID 8


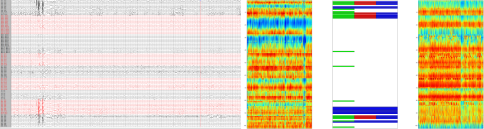


Patients ID 9


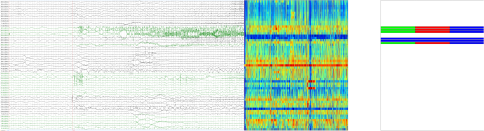


Patients ID 10


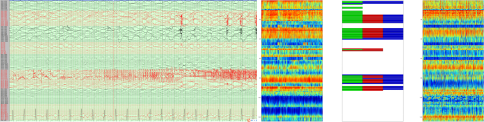


Patients ID 11


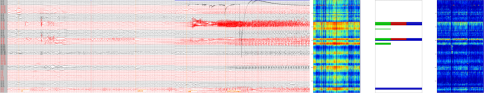


Patients ID 12


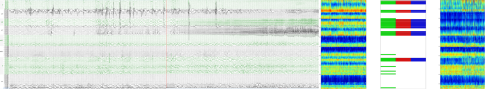


Patients ID 13


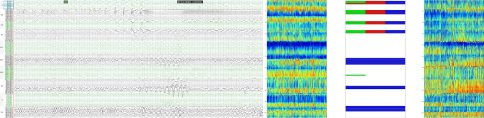


Patients ID 14


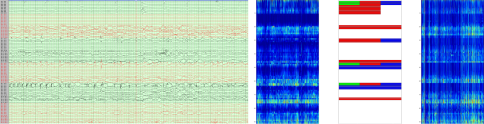


Patients ID 15


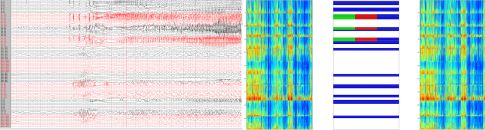


Patients ID 16


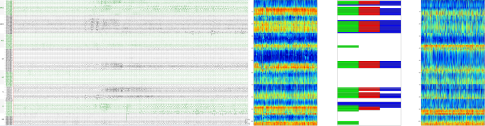


Patients ID 17


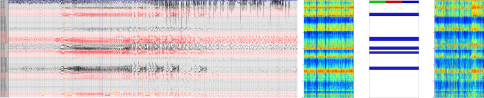


Patients ID 18


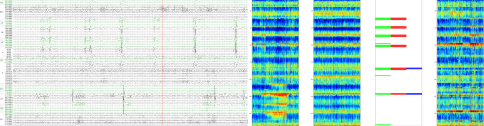

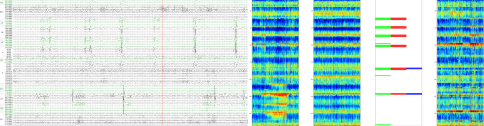


Patients ID 19


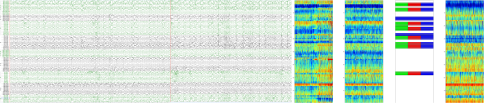


Patients ID 20


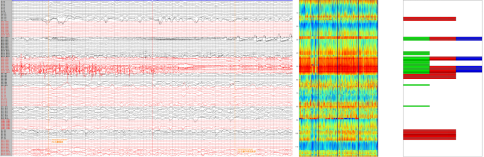


Patients ID 21


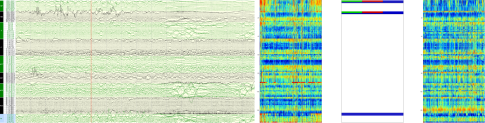


Patients ID 22


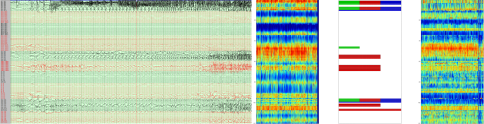


Patients ID 23


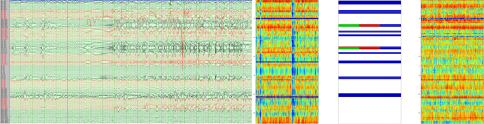


Patients ID 24


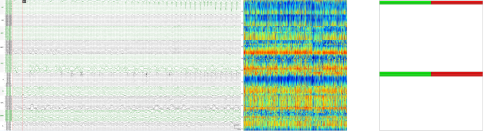


Patients ID 25


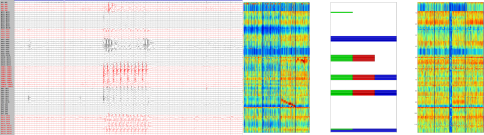


Patients ID 26


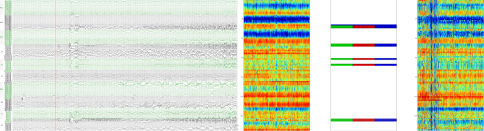


Patients ID 27


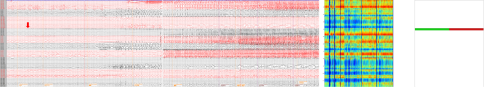


Patients ID 28


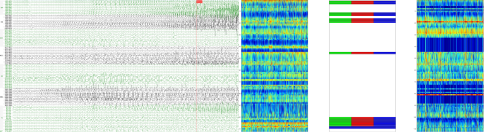


Patients ID 29


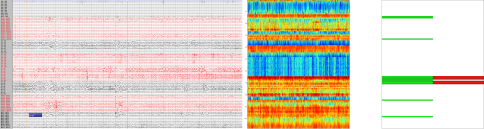


Patients ID 30


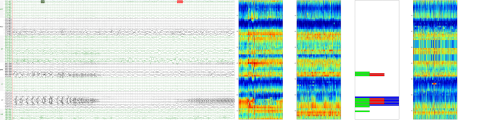

Supplement: Supplemental Information 2 [file peerj-13-19548-s002.docx]
